# STRATEGIES TO INCREASE MINORITY PARTICIPATION IN A HEALTHY AGING AND NEIGHBORHOOD STUDY

**DOI:** 10.1093/geroni/igz038.528

**Published:** 2019-11-08

**Authors:** Linda C Churchill, Rachel Siden, Annabella Aguirre, Marline Ruiz, Jie Cheng, Anthony Clarke, Kevin Kane, Wenjun Li

**Affiliations:** University of Massachusetts Medical School, Worcester, Massachusetts, United States

## Abstract

Adequate minority participation is critical to health disparity research. Conventional direct mails are less effective in minority recruitment. The Healthy Aging and Neighborhood Study developed a multifaceted, community-engaged, culturally and linguistically appropriate method to recruit community-living older adults (≥65y) in Worcester County, Massachusetts. The research team is bilingual, racially and culturally diverse. A direct mail campaign was conducted in a geographically diverse random sample of residents from neighborhoods with high concentrations of minorities, stratified by rurality. To increase minority participation, the mailings included an invitational letter or a flyer with a graphic that portrays diverse racial/ethnic background. We engaged communities by presenting the study at senior and community centers, and faith-based organizations that are frequented by minorities and by posting study information in minority social media groups (e.g., Chinese resident associations). Participants promoted the study to friends while staff promoted through professional or social networks. To recruit non-English speaking minorities, all materials were printed in age-friendly large fonts in Spanish or Chinese, and interviews were conducted using their preferred language. Within 9 months, we enrolled 326 participants, including 216 Whites, 57 Hispanics, 21 Blacks, 31 Asians, and 1 Native American. An additional 38 Asians are on the waiting list. Blacks were more likely to respond to community presentations. Hispanics were most likely to respond to the colorful flyer. Older minorities (>76y) were more likely to respond to presentations (57%) while the younger (<75y) to the mailings (60%). In summary, this multifaceted recruitment approach is effective in minority recruitment.

